# Improving GRPR-targeting peptides for radiotheranostics application: insights from chelator modifications and α-methyl-L tryptophan substitution

**DOI:** 10.1186/s41181-025-00402-2

**Published:** 2025-11-13

**Authors:** Karim Obeid, Ekaterina Bezverkhniaia, Vladimir Tolmachev, Anna Orlova, Panagiotis Kanellopoulos

**Affiliations:** 1 Department of Medicinal Chemistry, Uppsala University, 75183 Uppsala, Sweden; 2 Department of Immunology, Genetics and Pathology, Uppsala University, 75183 Uppsala, Sweden; 3 Science for Life Laboratory, Uppsala University, 75237 Uppsala, Sweden; 4 Department of Medicinal Chemistry, Uppsala University, 75123 Uppsala, Sweden

## Abstract

**Background:**

Targeting the gastrin-releasing peptide receptor (GRPR) is a promising approach for radionuclide therapy in prostate and breast cancers. GRPR-targeting peptides often have limited metabolic stability, which can compromise their clinical efficacy due to rapid degradation in the bloodstream, leading to reduced tumor uptake. We previously reported the GRPR-targeting peptide AU-RM26-M2 (DOTAGA-PEG_2_-Pip-[Sar^11^]RM26), which demonstrated promising pharmacokinetics in GRPR-expressing xenografts. In this study, we aimed to enhance the metabolic stability and targeting properties of AU-RM26-M2 by incorporating α-methyl-L-tryptophan (MetTrp) at position 8 in the pharmacophore, and to investigate the influence of chelator choice (DOTAGA vs. DOTA) for labeling with Lu-177, a β-emitting therapeutic nuclide.

**Results:**

Therefore, we designed two peptides: PKB2 (DOTAGA-PEG_2_-Pip-[MetTrp^8^, Sar^11^]RM26) and PKB3 (DOTA-PEG_2_-Pip-[MetTrp^8^, Sar^11^]RM26). For comparison, we also evaluated the DOTA-bearing analogue of AU-RM26-M2, PKB1 (DOTA-PEG_2_-Pip-[Sar^11^]RM26). PKB1, PKB2, and PKB3 were labeled with Lu-177, achieving high radiochemical yields (> 97%) and purities (> 93%). In PC-3 cells, [^177^Lu]Lu-PKB1, [^177^Lu]Lu-PKB2, and [^177^Lu]Lu-PKB3 showed affinity in the sub-nanomolar range and high specificity for GRPR, with a slow internalization rate. The radiopeptides with MetTrp^8^ modification had high metabolic stability against peptidases in vivo. In PC-3 xenografts, [^177^Lu]Lu-PKB2 and [^177^Lu]Lu-PKB3 demonstrated rapid background clearance and high GRPR-mediated tumor activity uptake at 2 h pi, exceeding activity uptake in the kidneys. Activity uptake in the tumor was highly retained at 24 h pi.

**Conclusions:**

This study led to the development of two metabolically stable GRPR-targeting radiopeptides, [^177^Lu]Lu-PKB2 and [^177^Lu]Lu-PKB3, with a high potential for targeted radionuclide therapy.

**Supplementary Information:**

The online version contains supplementary material available at 10.1186/s41181-025-00402-2.

## Introduction

Targeted radionuclide therapy (TRT) is an emerging field in nuclear medicine that offers a personalized treatment option for patients who do not respond to conventional chemotherapy. TRT allows delivery of cytotoxic radionuclides specifically to tumor cells, sparing healthy tissues from radiation-induced damage (Ersahin et al. 2011; Gill et al. 2017). This strategy has proven effectiveness in neuroendocrine tumors and prostate cancer, as demonstrated by the successful treatment using the radiolabeled somatostatin analog [^177^Lu]Lu-DOTA-TATE and prostate-specific membrane antigen inhibitor [^177^Lu]Lu-PSMA-617, respectively (Hennrich and Kopka 2019; Hennrich and Eder 2022).

The gastrin-releasing peptide receptor (GRPR) is a promising target for TRT. It is overexpressed in several malignancies, including prostate and breast cancers (Baun et al. 2024; Patel et al. 2006; Reubi et al. 2002). Although no GRPR-targeting radiopharmaceutical has been approved for clinical use yet, several therapeutic GRPR-targeting peptides have been evaluated in early-phase clinical trials. For example, human dosimetry studies have demonstrated that therapeutically relevant radiation absorbed doses to tumors can be safely achieved in patients with castration-resistant prostate cancer using [^177^Lu]Lu-RM2 and [^177^Lu]Lu-AMTG (Kurth et al. 2020, 2024). [^177^Lu]Lu-NeoB is currently undergoing phase 1 and 2 clinical trials for the therapy of patients with GRPR-expressing tumors (Lu-NeoB 2025). These clinical studies underline the growing interest in the GRPR as a target for TRT.

The most studied GRPR-targeting radiopharmaceuticals are peptide-based. Such peptides offer several advantages, including low production costs, high target specificity, and rapid clearance from non-target tissues, rapid tissue penetration, and low immunogenicity (Chakraborty et al. 2023). Initial GRPR-targeting peptides were bombesin (BBN)-based agonists. Their clinical translation was hindered by acute adverse effects (Bodei et al. 2007). This led to a shift toward GRPR antagonists, which offer better pharmacokinetic profiles and little to none observed side effects due to the activation of the receptor after administration in patients. (Mansi et al. 2021 ; Baratto et al. 2018; Nock et al. 2023). However, a major challenge in developing GRPR-targeting peptides lies in their poor metabolic stability. These peptides are rapidly degraded in the bloodstream by proteolytic enzymes, particularly by the neutral endopeptidase (NEP) (Shipp et al. 1991). NEP is known to cleave GRPR antagonists at vulnerable sites, such as Gly^11^-His^12^ and Gln^7^-Trp^8^. The enzymatic degradation significantly reduces the concentration of peptides in blood and hinders their ability to reach their target sites in tumors (Lymperis et al. 2018; Teufel et al. 2003; Linder et al. 2009). Incorporation of unnatural amino acids at known cleavage sites is a well-known strategy to prevent their recognition by proteolytic enzymes, thereby enhancing resistance to enzymatic degradation (Evans et al. 2020). For instance, the replacement of Gly^11^ with Sar^11^ (Sar: sarcosine, N-methylglycine) resulted in [^111^In]In-AU-RM26-M1 and [^99m^Tc]Tc-DB15 with improved stability towards NEP (Abouzayed et al. 2023; Nock et al. 2021). Prior studies have also demonstrated that α-methyl-L-tryptophan (MetTrp) at position 8 of [^177^Lu]Lu-RM2 protects the peptide from enzymatic degradation and improves its in vivo performance (Günther et al. 2022). Modifications in small peptides can be challenging as they might alter affinity to the receptor as well as the peptide´s pharmacokinetics (Mansi et al. 2021). This was evident with [^111^In]In-SB4, where the substitution of Gly^11^ with DAla^11^ enhanced the peptide´s half-life, but lowered GRPR affinity compared to the parent compound [^111^In]In-SB3 (Lymperis et al. 2019).

**Fig. 1:**
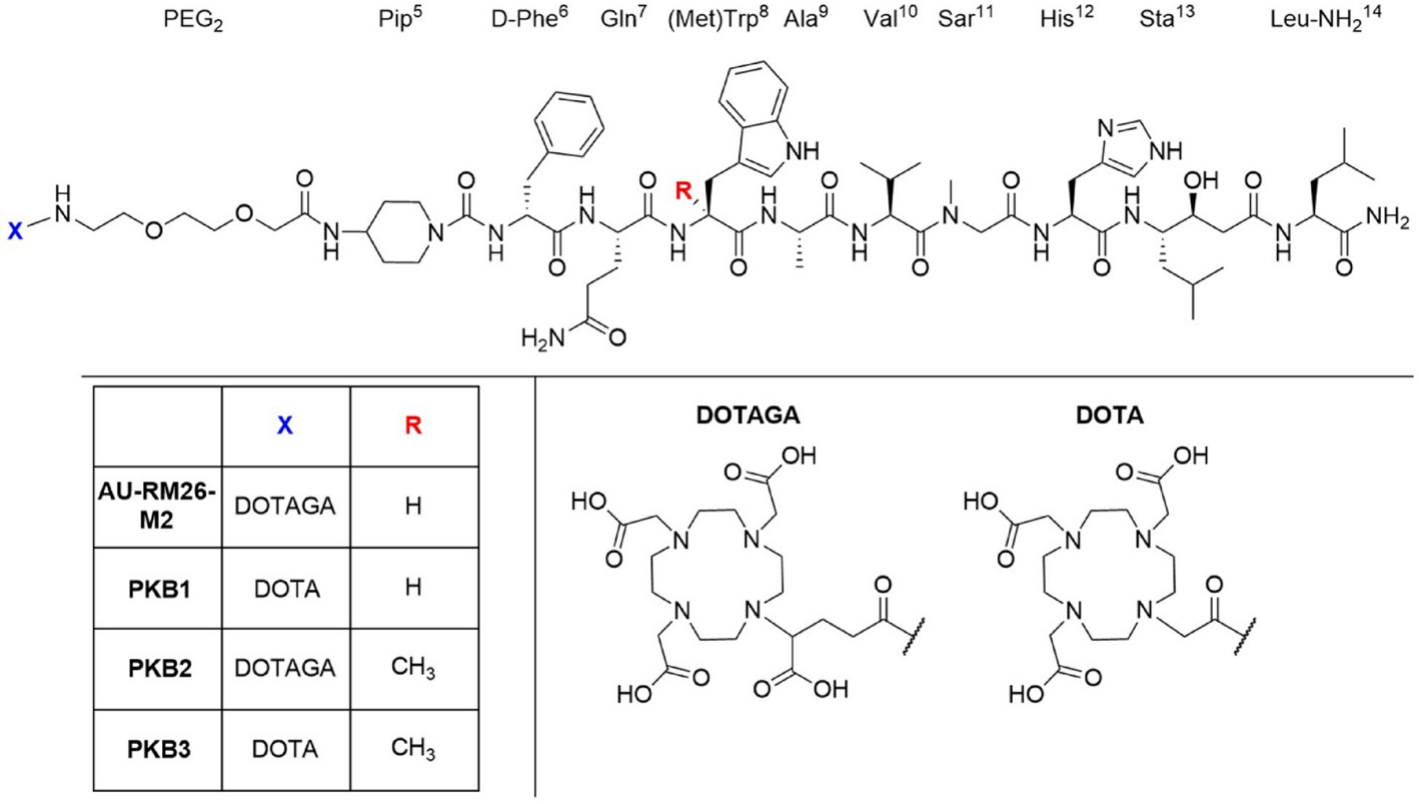
Chemical structures of AU-RM26-M2 [DOTAGA-PEG_2_-Pip-[Sar^11^]RM26]; PKB1 [DOTA-PEG_2_-Pip-[Sar^11^]RM26], PKB2 [DOTAGA-PEG_2_-Pip-[MetTrp^8^, Sar^11^]RM26], and PKB3 [DOTA-PEG_2_-Pip-[MetTrp^8^, Sar^11^]RM26]. DOTAGA: (1,4,7,10-tetra(carboxymethyl)-1,4,7,10-tetraazacyclo-dodecane-1-glutaric acid); DOTA: 1,4,7,10-tetraazacyclododecane-1,4,7,10-tetraacetic acid]; Pip: 4-amino-1-carboxymethyl-piperidine; PEG_2_:8-amino-3,6 dioxa-octanoic acid; RM26: D-Phe^6^-Gln^7^-Trp^8^-Ala^9^-Val^10^-Gly^11^-His^12^-Sta^13^-Leu^14^-NH_2_

We previously investigated a series of peptides based on the RM26 scaffold (D-Phe^6^-Gln^7^-Trp^8^-Ala^9^-Val^10^-Gly^11^-His^12^-Sta^13^-Leu^14^-NH_2_). Among them, AU-RM26-M2 (DOTAGA-PEG_2_-Pip-[Sar^11^]RM26) showed a fast background clearance and high metabolic stability (76% intact peptide detected in peripheral mouse blood 5 min pi) (Kanellopoulos et al. 2025). To further increase in vivo stability and improve tumor-targeting properties, we investigated the influence of incorporating MetTrp^8^ in the pharmacophore of AU-RM26-M2. Additionally, we substituted the DOTAGA with the DOTA chelator to evaluate its impact on biodistribution and receptor interaction. Therefore, we designed two new variants of GRPR antagonists bearing the modification mentioned above at position 8: PKB2 (DOTAGA-PEG_2_-Pip-[MetTrp^8^, Sar^11^]RM26) and PKB3 (DOTA-PEG_2_-Pip-[MetTrp^8^, Sar^11^]RM26) with AU-RM26-M2 and its DOTA-coupled variant, PKB1 (DOTA-PEG_2_-Pip-[Sar^11^]RM26) serving as comparators (Fig. 1). PKB2 and PKB3 were labeled with Lu-177 and evaluated in vitro and in vivo using GRPR-positive PC-3 cells.

## Materials and methods

### General

The peptides AU-RM26-M2, PKB1, PKB2, PKB3, and NOTA-PEG_2_-RM26 were custom-synthesized by Pepmic Co., Ltd. (Suzhou, China) (Kanellopoulos et al. 2025; Obeid et al. 2024). [^177^Lu]LuCl_3_ was purchased from ITM Isotope Technologies (Munich, Germany). The GRPR-expressing PC-3 human prostate cancer cell line was purchased from American Type Culture Collection (Manassas, VA, USA). Cells were cultured using Roswell Park Memorial Institute (RPMI) 1640 medium (containing L-glutamine), supplemented with 10% fetal bovine serum and 1% penicillin–streptomycin (100 IU/mL penicillin and 100 µg/mL streptomycin). Cell detachment was performed using 0.25% Trypsin–EDTA. Both media supplements and trypsin were provided by Biochrom AG (Berlin, Germany). The cells were maintained at 37 °C in a humidified 5% CO₂ atmosphere. The activity content of all collected samples was measured using the Wizard2TM gamma counter (Revvity, Hägersten, Sweden).

### Radiolabeling

Labeling of AU-RM26-M2, PKB1, PKB2, and PKB3 with Lu-177 was performed by adding 5–20 µL (50–70 MBq) [^177^Lu]LuCl_3_ to 5 µL peptide (1 mM), 20 µL EtOH (99.5%), and 40 µL 0.2 M ammonium acetate pH 5.5. After mixing, the labeling solution was incubated for 30 min at 85 °C. For the in vivo stability, the labeling protocol was slightly changed: 10 µL peptide were added to 10 µL ascorbic acid (0.1 M), 20 µL ethanol, 80 µL 0.5 M ammonium acetate pH 5.5, and 100–30 µL of [^177^Lu]LuCl_3_ (~ 230 MBq).

The radiochemical purity of [^177^Lu]Lu-PKB1, [^177^Lu]Lu-PKB2, and [^177^Lu]Lu-PKB3 was determined using reverse-phase high-protein liquid chromatography (HPLC). The HPLC system included: LaPrep Sigma HPLC LP1100 pump (Hitachi High-Tech Corporation, Hitachinaka, Ibaraki, Japan), a 40D LWL UV detector with a 4 µL flow cell (Knauer, Berlin, Germany), a flow-scan radioactivity detector (Bioscan) with an FC-3300 NaI/PMT radioactivity probe (Eckert & Ziegler, Berlin, Germany), a manual Rheodyne 7725i injector fitted with a 20 µL loop (IDEX Health & Science, LLC, Rohnert Park, CA, USA), and a Luna Omega C18 column was used (5 μm, 100 Å, 100 × 4.6 mm, Phenomenex, Værløse, Denmark). The elution method used started at 95% 0.1% v/v aqueous trifluoroacetic acid (TFA) / 5% 0.1% v/v TFA in acetonitrile (MeCN), and over 20 min, it reached 40% TFA/60% MeCN.

Radiochemical yields of [^177^Lu]Lu-PKB1 (n = 7), [^177^Lu]Lu-PKB2 (n = 7), [^177^Lu]Lu-PKB3 (n = 7) and [^177^Lu]Lu-AU-RM26-M2 (n = 2) were determined by instant thin-layer chromatography (iTLC) using silica gel-impregnated glass microfiber strips (Agilent Technologies, Santa Clara, CA, USA) as the stationary phase and 0.2 M citric acid as the mobile phase. The iTLC yields were evaluated using Cyclone® Plus Phosphorimager (Revvity, Hägersten, Sweden).

### In vitro studies

For the in vitro GRPR specificity assay of [^177^Lu]Lu-PKB1, [^177^Lu]Lu-PKB2, and [^177^Lu]Lu-PKB3, 8 × 10^5^ PC-3 cells/well were seeded in 6-well plates. After discarding the medium, the cells were washed with cold (4 °C) phosphate-buffered saline containing 1% w/v bovine serum albumin (PBS/BSA). The radiopeptides (C_f_ = 1 nM in 1 mL PBS/BSA) were added to all wells. A subset of wells was also treated with an excess of unlabeled GRPR-binding peptide NOTA-PEG_2_-RM26 (Bjäreback et al. 2025) (C_f_ = 1 µM in 1 mL PBS/BSA) to block GRPR. After incubation for 1 h at 37 °C, the supernatant was removed, and the cells were washed and then collected by treating them with 1 M NaOH. The activity of the collected samples was measured with the gamma counter. This assay was performed in triplicate for each radiopeptide.

For cellular processing evaluation, 8 × 10^5^ PC-3 cells were seeded in 35 mm Petri dishes. After removing the medium and washing the cells with PBS/BSA, the test radiopeptide was added to the dishes (C_f_ = 1 nM), and the dishes were incubated at 37 °C. At predetermined time points (1 h, 4 h, and 24 h), the cells were treated with 500 µL of acidic solution (0.2 M glycine buffer with 0.15 M NaCl and 4 M urea, pH 2) and incubated on ice for 5 min; and the supernatant was collected (membrane-bound fraction). The cells were washed with 500 µL of the same acid buffer and collected. The cells were then washed with PBS/BSA and treated with 1 M NaOH, incubated for 15 min at 37 °C, and collected (internalized fraction). The activity of the samples was measured with the gamma counter. Three repetitions were performed for each time point.

For these two experiments, statistical analysis was performed with GraphPad Prism 10 using an unpaired two-tailed t-test.

The GRPR affinity of the radiopeptides was determined using the LigandTracer white (Ridgeview Instruments AB, Uppsala, Sweden). For this purpose, 3 × 10^6^ PC-3 cells were seeded in 100 mm Petri dishes (Corning Incorporated, New York, USA). The radiopeptides were tested at 1 nM and 5 nM concentrations to get the association curves. After reaching the plateau, the radioactive medium was replaced with fresh complete media to determine the dissociation curve. All runs were done at room temperature (22–25 °C) The sensorgrams were analyzed with TraceDrawer (Ridgeview Instruments AB, Uppsala, Sweden), the curves were fitted with a 1:2 model, and the dissociation constants (K_D_ values) were calculated. Sensorgrams from the LigandTracer analysis can be found in the supplementary material (Figures S1-S3).

### In vivo studies

The in vivo experiments were carried out following European standards for laboratory animal welfare. They received approval from the regional ethics committee for animal research in Uppsala, Sweden (permit number 00473/21).

The in vivo stability of [^177^Lu]Lu-PKB1, [^177^Lu]Lu-PKB2, and [^177^Lu]Lu-PKB3 was evaluated in healthy NMRI mice. Two animals per peptide were injected intravenously with a bolus of test radioligand (50 MBq, 2 nmol of peptide in 100 μL PBS/EtOH v/v 9:1) – Control group. To inhibit NEP, additional groups of two mice per compound were orally treated with 12 mg Entresto® (Novartis, Basel, Switzerland) 30 min before receiving the intravenous injection—Entresto group. Five minutes post-injection (pi), the animals were euthanized, and the blood was collected and immediately mixed with EDTA (0.1 mM, 40 μL) in Eppendorf tubes on ice. The mixture was spun at 2000 g for 10 min at 4 °C in an Eppendorf Centrifuge 5430 R (Eppendorf AG, Hamburg, Germany), and the plasma was collected and diluted with an equal volume of MeCN (4 °C). After a second centrifugation at 15,000 g and 4 °C for 10 min, the supernatant was collected in an Eppendorf tube and concentrated to 50–100 μL at 50 °C using a mild stream of N_2_. PBS was added to the samples to reach a final volume of 200 μL, and the solutions were filtered through a syringe filter (0.22 μm, 13 mm, VWR International, Radnor, USA). The filtered solution was analyzed by reversed-phase radio-HPLC to determine the percentage of intact peptides. The peak corresponding to the intact peptide was identified by injection of an aliquot of the labeling solution. Representative radiochromatograms for the four radioligands without (Control) and with pre-treatment with Entresto (Entresto) are given in the supplementary material (Figures S4-S11).

The biodistribution profile of [^177^Lu]Lu-PKB2 and [^177^Lu]Lu-PKB3 was evaluated at 2, 4, and 24 h pi time points on Balb/c nu/nu mice. Each mouse was xenografted with a subcutaneous injection of 8 × 10⁶ PC-3 cells in the right hind leg. After approximately 4 weeks, the animals were injected into the tail vein with 100 µL of radiopeptide (100 pmol—110–130 kBq, diluted in PBS/BSA). Four animals were used for each time point per peptide. Two additional groups of 3 mice each were also co-injected with a 100-fold molar excess of unlabeled NOTA-PEG_2_-RM26 (Bjäreback et al. 2025) and sacrificed at 2 h pi to demonstrate in vivo GRPR specificity of both radiopeptides. At the determined time points, the mice were euthanized, organs were collected and weighed, and their activity content was measured in the gamma counter. Results were presented as the percentage of injected activity per gram (%IA/g) for the collected organs, and the percentage of injected activity (%IA) for the gastrointestinal tract (GI) and the carcass. Biodistribution at 4 h pi of [^177^Lu]Lu-AU-RM26-M2 was also performed as a comparator in the same way described above. Statistical analysis was performed with GraphPad Prism 10 using two way ANOVA with Tuckey’s or Šidák’a post hoc analysis.

## Results

### Radiolabeling

**Fig. 2:**
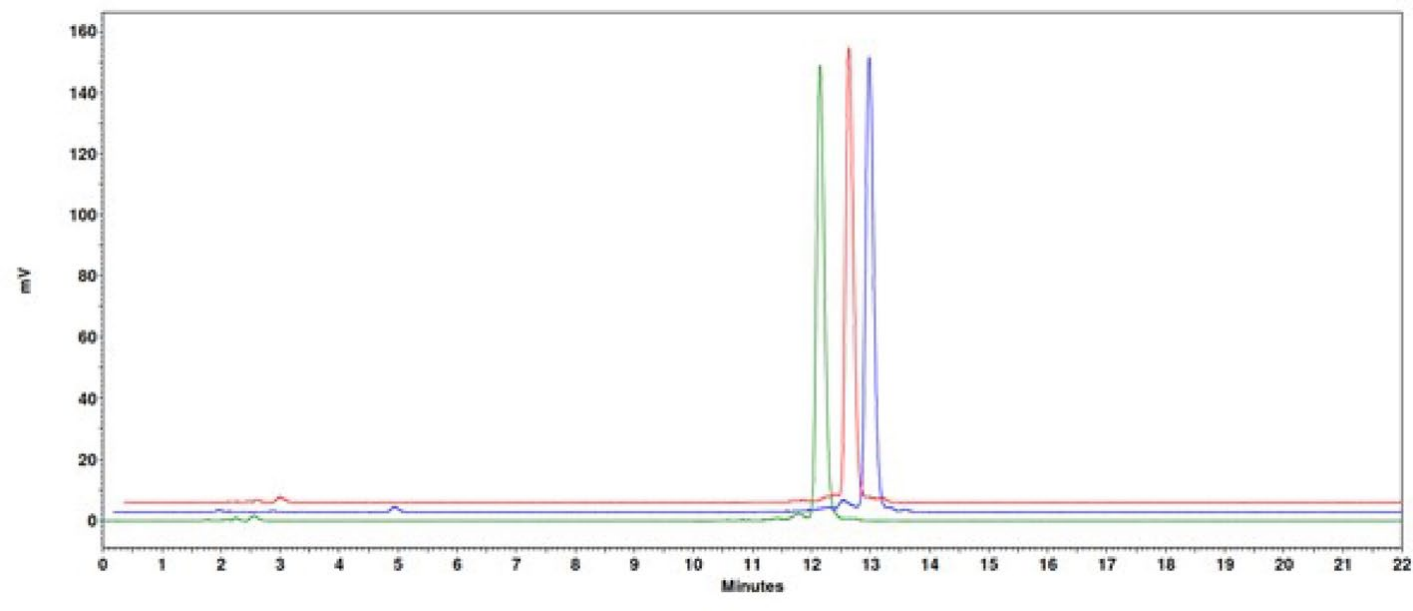
Representative radiochromatograms of the labeling solutions of [^177^Lu]Lu-PKB1 (green), [^177^Lu]Lu-PKB2 (blue), and [^177^Lu]Lu-PKB3 (red)

High radiochemical yields (> 97%) were observed with iTLC analysis for [^177^Lu]Lu-AU-RM26-M2 (n = 2), [^177^Lu]Lu-PKB1 (n = 7), [^177^Lu]Lu-PKB2 (n = 7), and [^177^Lu]Lu-PKB3 (n = 7). The radiochemical purity determined by HPLC for [^177^Lu]Lu-PKB1, [^177^Lu]Lu-PKB2, and [^177^Lu]Lu-PKB3 was > 93% (Fig. 2).

### In vitro studies

**Fig. 3:**
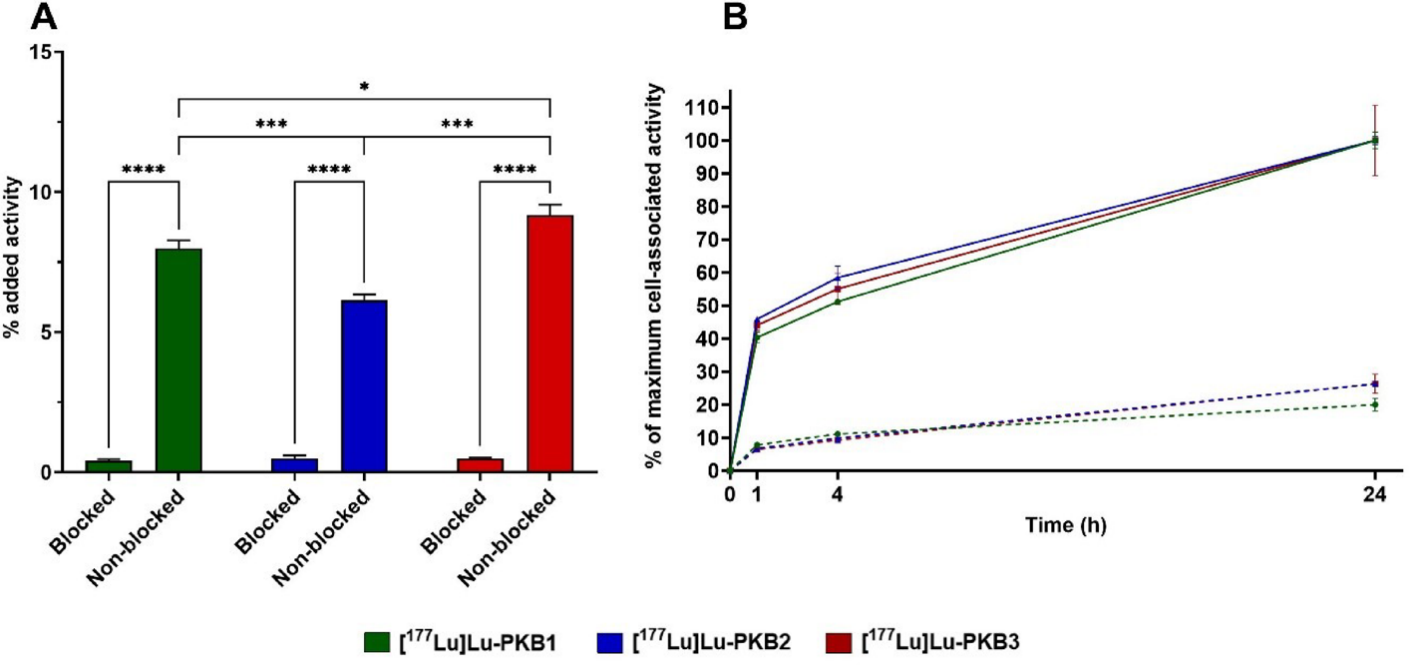
In vitro binding specificity (**A**) and cellular processing (**B**) of [^177^Lu]Lu-PKB1, [^177^Lu]Lu-PKB2, and [^177^Lu]Lu-PKB3 in PC-3 cells at 37 °C and 1 nM final concentration of peptide. Some error bars may not be visible due to minimal variability. Solid line: total cell-associated uptake (membrane bound + internalized fraction); dashed line: internalized fraction. Data are presented as mean ± SD (n = 3). **** corresponds to *p* < 0.0001, *** to *p* < 0.001, and * to *p* < 0.05

The in vitro specificity and cellular processing experiments were done in parallel to allow head-to-head comparisons. [^177^Lu]Lu-PKB1, [^177^Lu]Lu-PKB2 and [^177^Lu]Lu-PKB3 showed a GRPR-mediated activity uptake in PC-3 cells (Fig. 3A). The total cellular activity uptake was the highest for [^177^Lu]Lu-PKB3, followed by [^177^Lu]Lu-PKB1, and the lowest uptake was found for [^177^Lu]Lu-PKB2 (all differences were significant as shown in Fig. 3A). The three radiopeptides had a similar pattern of cellular uptake with rapid binding and a slow internalization (Fig. 3B). After 24 h of continuous incubation, 20–26% of the total cell-associated activity (membrane bound + internalized fraction) was internalized for all three tested radiopeptides.

The affinity to GRPR was analyzed using a 1:2 model interaction because it was found that GRPR antagonists are involved in two types of interactions with the receptor (Xu et al. 2013). The K_D_ values of [^177^Lu]Lu-PKB1, [^177^Lu]Lu-PKB2, and [^177^Lu]Lu-PKB3 were similar (Table 1), demonstrating a high affinity towards GRPR. K_D1_ and K_D2_ values were in sub-nanomolar ranges.

**Table 1:** Kinetic measurements using LigantTracer for [^177^Lu]Lu-AU-RM26-M2, [^177^Lu]Lu-PKB1, [^177^Lu]Lu-PKB2, [^177^Lu]Lu-PKB3 in PC-3 cells

Interaction constants	[^177^Lu]Lu-AU-RM26-M2*	[^177^Lu]Lu-PKB1	[^177^Lu]Lu-PKB2	[^177^Lu]Lu-PKB3
k_a1_ (1/(M*s))	1.84 × 10^5^	9.80 × 10^4^	1.12 × 10^5^	1.30 × 10^5^
k_d1_ (1/s)	3.53 × 10^–5^	5.76 × 10^–6^	1.55 × 10^−5^	7.56 × 10^−6^
K_D1_ (M)	1.92 × 10^–10^	5.88 × 10^–11^	1.38 × 10^−10^	5.82 × 10^−11^
k_a2_ (1/(M*s))	1.08 × 10^6^	4.16 × 10^5^	1.25 × 10^5^	2.04 × 10^5^
k_d2_ (1/s)	5.94 × 10^–2^	2.21 × 10^–4^	4.23 × 10^−4^	2.12 × 10^–4^
K_D2_ (M)	5.52 × 10^–8^	5.32 × 10^–10^	3.38 × 10^−9^	1.04 × 10^−9^
B_max1_ / B_max2_	0.16	1.42	0.41	0.82

For the association phase 2 concentrations were used (1 and 5 nM) followed by the dissociation phase

Ka, association constant; kd, dissociation constant; K_D_, equilibrium dissociation constant

*Values are taken from previously published data (25)

### In vivo studies

**Fig. 4:**
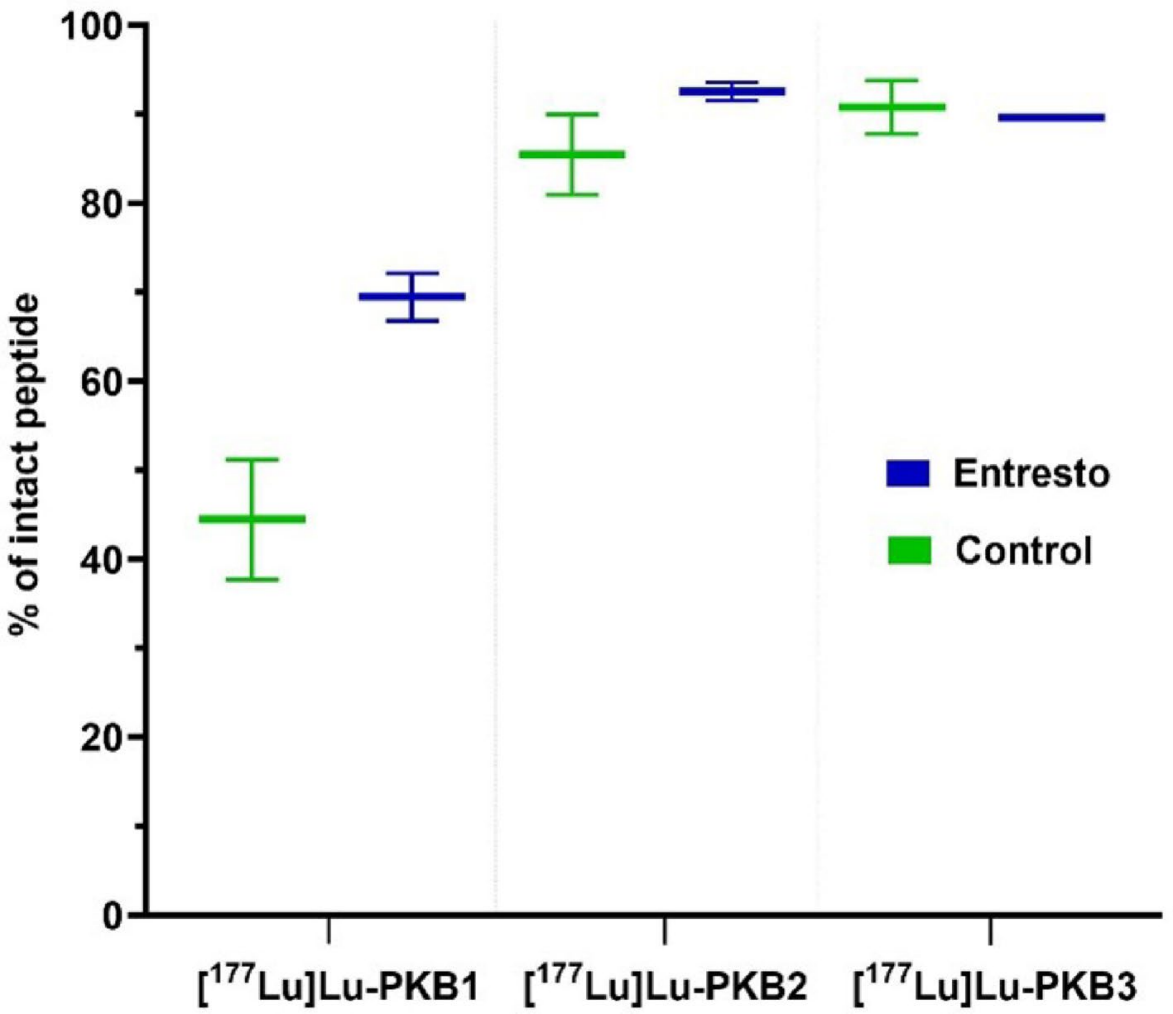
Percentage of the intact [^177^Lu]Lu-PKB1, [^177^Lu]Lu-PKB2, and [^177^Lu]Lu-PKB3 detected in peripheral NMRI mice blood, 5 min pi, determined by radio-HPLC analysis. Control: group injected with the radiopeptide only; Entresto: group orally pre-treated with NEP inhibitor 30 min before injection of the radioligand. Data are presented as the mean of two values per group (n = 2); error bars correspond to the minimum and maximum values recorded. The error bar of [^177^Lu]Lu-PKB3 (Entresto) is not visible due to minimal variability

The in vivo stability of the radiopeptides in blood circulation 5 min pi revealed some differences among the peptides (Fig. 4, Table S1). [^177^Lu]Lu-PKB1 had the lowest metabolic stability compared to the [^177^Lu]Lu-PKB2 and [^177^Lu]Lu-PKB3, but showed a marked improvement with Entresto pre-treatment. In contrast, both [^177^Lu]Lu-PKB2 and [^177^Lu]Lu-PKB3 demonstrated high intrinsic stability. While [^177^Lu]Lu-PKB2 demonstrated minimal degradation by NEP, [^177^Lu]Lu-PKB3 remained highly stable and showed no signs of NEP-mediated degradation.

**Fig. 5:**
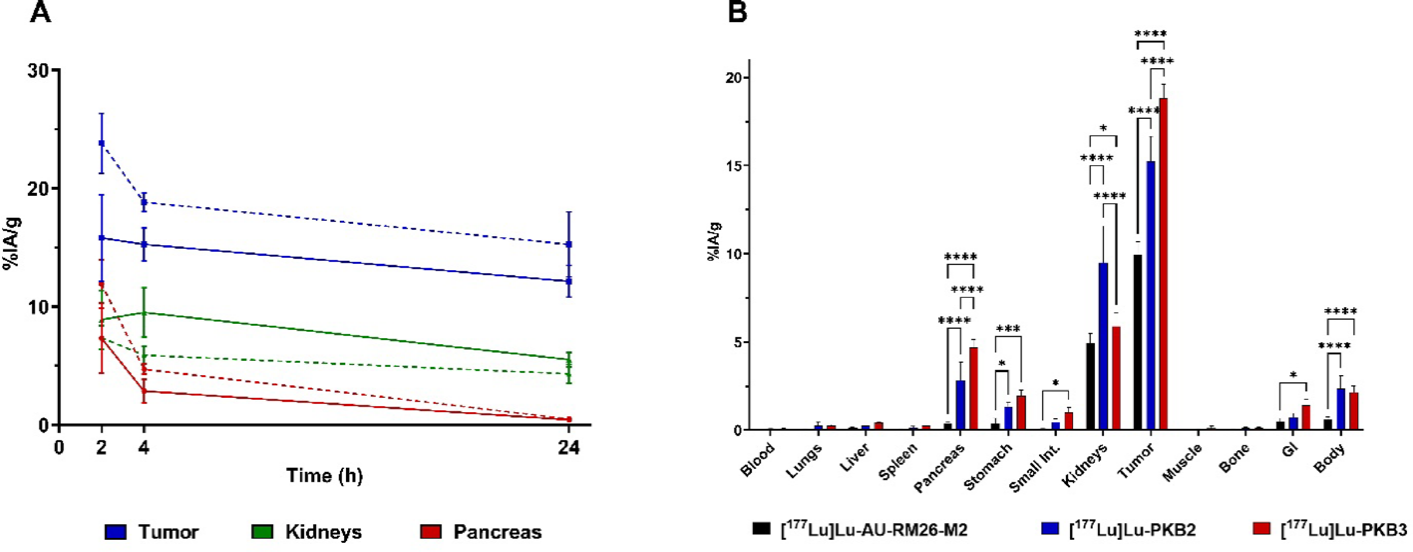
**A** Clearance of [^177^Lu]Lu-PKB2 (full line) and [^177^Lu]Lu-PKB3 (dashed line) from tumors, kidneys, and pancreas. Statistical differences at 2 h tumor (*p* < 0.0001), pancreas (*p* < 0.0001). Statistical differences at 4 h: tumor (*p* < 0.0001), kidneys (*p* < 0.0001), pancreas (*p* < 0.0001). No statistical differences were observed at 24 h pi. **B** Biodistribution of [^177^Lu]Lu-PKB2, [^177^Lu]Lu-PKB3 compared with [^177^Lu]Lu-AU-RM26-M2 at 4 h pi. All statistical comparisons are made against [^177^Lu]Lu-AU-RM26-M2 (black). * corresponds to *p* < 0.05; ** to *p* < 0.01; *** to *p* < 0.001 and **** to *p* < 0.0001. Statistical analysis was performed using two-way ANOVA with Tuckey’s post hoc analysis or when two groups were compared (2 h and 24 h pi) Šidák post hoc analysis was used instead

[^177^Lu]Lu-PKB1 was excluded from biodistribution studies in tumor-bearing mice due to its low metabolic stability. Biodistribution studies for [^177^Lu]Lu-PKB2 and [^177^Lu]Lu-PKB3 were performed without NEP inhibitor pre-treatment due to their high metabolic stability (Tables S2 and S3). Both [^177^Lu]Lu-PKB2 and [^177^Lu]Lu-PKB3 showed fast clearance from non-target organs, including blood, lungs, spleen, muscle, and bone; the activity uptake in the mentioned organs was < 0.6%IA/g at 2 h pi. Minimal activity uptake was observed in the stomach and small intestines (organs with endogenous GRPR expression) at 2 h pi (< 2.5%IA/g), with further clearance at 4 h pi. At 2 h pi. [^177^Lu]Lu-PKB3 had significantly higher activity uptake in the pancreas (organ with elevated endogenous GRPR expression) than [^177^Lu]Lu-PKB2 at 2 h pi, which rapidly washed out at the later time points (Fig. 5A). [^177^Lu]Lu-PKB3 showed a lower kidney activity uptake, although the differences were not statistically significant(Fig. 5A). [^177^Lu]Lu-PKB3 showed higher hepatobiliary excretion than [^177^Lu]Lu-PKB2, nevertheless, activity uptake was below 2%IA for the GI with content at all time points. Both radiopeptides had high activity uptake in tumors, with [^177^Lu]Lu-PKB3 showing a 1.5-fold higher activity uptake compared to [^177^Lu]Lu-PKB2 at 2 h pi. Both [^177^Lu]Lu-PKB2 and [^177^Lu]Lu-PKB3 retained high tumor activity uptake even at the 24 h time point. At 2 h pi, tumor activity uptake was 122-fold higher than blood for [^177^Lu]Lu-PKB2 and 109-fold higher for [^177^Lu]Lu-PKB3. For [^177^Lu]Lu-PKB3, activity uptake in tumors was 3.2-fold higher than in kidneys at 2 h pi, while for [^177^Lu]Lu-PKB2 the ratio was 1.8. Statistical differences of kidneys, tumor, and pancreas are noted in Fig. 5A legend.

The biodistribution of the parent compound [^177^Lu]Lu-AU-RM26-M2 was performed at 4 h pi for comparison (Fig. 5B). [^177^Lu]Lu-PKB2 and [^177^Lu]Lu-PKB3 demonstrated significantly higher tumor and pancreas uptake than [^177^Lu]Lu-AU-RM26-M2. Kidney activity uptake of [^177^Lu]Lu-PKB3 was comparable to that of [^177^Lu]Lu-AU-RM26-M2, while [^177^Lu]Lu-PKB2 showed twofold higher kidney activity uptake than the parent compound.

Co-injection of a 100-fold molar excess of unlabeled NOTA-PEG_2_-RM26 significantly (*p* < 0.0001) reduced tumor and pancreas activity uptake of [^177^Lu]Lu-PKB2 and [^177^Lu]Lu-PKB3 and at 2 h pi, confirming the GRPR specificity of both peptides (Table S2 and S3).

## Discussion

TRT offers the potential for personalized cancer treatment strategies (Ersahin et al. 2011). The GRPR is an obvious molecular target for TRT due to its overexpression in various tumors and relatively low expression in healthy tissues (Sun et al. 2023). Early-phase clinical trials have confirmed the safety of targeting the GRPR with small BBN-based peptides for imaging and therapy (Kurth et al. 2020, 2024; Bjäreback et al. 2025; Chernov et al. 2023; Gruber et al. 2020; Bakker et al. 2021).

Peptides are promising vehicles for delivering radionuclides to tumors, but their rapid enzymatic degradation poses challenges in their design (Evans et al. 2020 May 14; Okarvi 2008). We have previously reported the GRPR-targeting peptide AU-RM26-M2, an improved analogue of DOTAGA-PEG_2_-RM26, with reasonably high in vivo stability (76% intact peptide detected in peripheral mouse blood 5 min pi) (Kanellopoulos et al. 2025). To further improve in vivo stability, we hypothesized that incorporating MetTrp⁸ into the AU-RM26-M2 pharmacophore would enhance its in vivo stability, an approach that was reported to increase the metabolic stability of other GRPR-targeting peptides (Günther et al. 2022). Additionally, we compared DOTAGA and DOTA chelators to evaluate their impact on GRPR affinity and tumor retention in order to choose the optimal one in combination with Lu-177, aiming for use in TRT.

The newly designed peptides, PKB2 and PKB3, as well as the new comparator PKB1, were labeled with Lu-177, achieving high radiochemical yields and purities, without the need for post-labeling purification, which is essential to simplify preparation and ensure reproducibility in a clinical setting. [^177^Lu]Lu-PKB1, [^177^Lu]Lu-PKB2, and [^177^Lu]Lu-PKB3 showed a fast GRPR-mediated uptake in PC-3 cells, with a slow internalization rate over time, a typical behavior of GRPR antagonists (Cescato et al. 2008). Interestingly, the DOTA-conjugated radiopeptides, [^177^Lu]Lu-PKB1 and [^177^Lu]Lu-PKB3, demonstrated a significantly (*p* < 0.001) higher uptake than the DOTAGA-conjugated peptide [^177^Lu]Lu-PKB2. This difference can be attributed to the different metal-chelate. Either the different conformation of the chelate and/or the more positive charge of the Lu-DOTA complex is more preferable for the interaction between the ligand and the receptor. From previous reports we know that positive charges at the N-terminus of GRPR antagonists are enhancing receptor affinity (Maina et al. 2017). This beneficial contribution of the charge and/or the metal-chelate, documented also in other reports (Kanellopoulos et al. 2020; Chatalic et al. 2016; Sano et al. 2004), is also represented in the affinity constants determined with the Ligand Tracer, where [^177^Lu]Lu-PKB1 and [^177^Lu]Lu-PKB3 had better K_D_ values than [^177^Lu]Lu-PKB2 and [^177^Lu]Lu-AU-RM26-M2 (Table 1) (Kanellopoulos et al. 2025). Nevertheless, the two new radioligands, [^177^Lu]Lu-PKB2 and [^177^Lu]Lu-PKB3 had K_D1_ and K_D2_ values in the picomolar and sub-nanomolar range respectively, indicating their high GRPR affinity. As expected, the incorporation of MetTrp^8^ did not affect GRPR affinity, indicating that this modification is well tolerated by the receptor, while still having a marked positive effect on metabolic stability (Fig. 4). Interestingly, [^177^Lu]Lu-PKB1 showed inferior stability with only 44% of intact peptide detected. This indicates that the choice of the metal-chelate can also influence in vivo stability, on top of the receptor affinity and overall pharmacokinetics. This indicates that the difference of the charge and/or the conformation the metal-chelate could alter the peptide´s conformation, reducing its accessibility to proteolytic enzymes. The metabolic stability of [^177^Lu]Lu-PKB1 and [^177^Lu]Lu-PKB2 improved upon in situ NEP inhibition, confirming NEP´s role in degrading GRPR-targeting radiopeptides, as observed in previous studies (Kanellopoulos et al. 2025; Obeid et al. 2024 ; Mitran et al. 2016, 2019). In addition, pre-treatment of the mice with Entresto did not result in increase of the amount of [^177^Lu]Lu-PKB3 detected intact, thus hinting its resistance to degradation byNEP. Despite the very strong indications and the quite good reproducibility of the data, there is a need to point to the small number of repetitions, thus hindering a robust statistical analysis and clear verdicts concerning small differences in metabolic stability by the radioligands tested.

Due to the poor in vivo stability of [^177^Lu]Lu-PKB1, biodistribution studies in mice bearing GRPR-expressing xenografts were performed for only [^177^Lu]Lu-PKB2 and [^177^Lu]Lu-PKB3 in comparison with [^177^Lu]Lu-AU-RM26-M2. [^177^Lu]Lu-PKB2 and [^177^Lu]Lu-PKB3 had fast clearance from non-target tissues (Fig. 5, Tables S2-S3), which is essential for TRT. [^177^Lu]Lu-PKB3 demonstrated 1.5-fold higher activity uptake in the tumor and pancreas (a GRPR-expressing organ (Varasteh et al. 2015)) compared to [^177^Lu]Lu-PKB2. This observation correlates with the higher cellular-uptake and better binding affinities for [^177^Lu]Lu-PKB3.

The activity in the pancreas was rapidly washed out at later time points for both [^177^Lu]Lu-PKB2 and [^177^Lu]Lu-PKB3. On the contrary, activity in tumors was strongly retained even at the 24 h time point, with [^177^Lu]Lu-PKB3 tending to have higher tumor activity uptake than [^177^Lu]Lu-PKB2 over time. [^177^Lu]Lu-PKB2 and [^177^Lu]Lu-PKB3 demonstrated high in vivo specificity, as evidenced by the significant reduction in activity uptake in the tumor and GRPR-expressing organs (pancreas, stomach, and small intestines (Xiao et al. 2001; Monstein 2006)) after co-administration of an excess of NOTA-PEG_2_-RM26.

[^177^Lu]Lu-PKB2 had higher kidney activity uptake than [^177^Lu]Lu-PKB3 at all time points. Tumor activity uptake at 24 h pi was 3.5-fold higher than in kidneys for [^177^Lu]Lu-PKB3 and 2.2-fold higher for [^177^Lu]Lu-PKB2. Activity uptake in the kidneys is essential as it is a common dose-limiting organ in TRT (Dawson et al. 2010). [^177^Lu]Lu-PKB3 showed twofold higher hepatic activity uptake than [^177^Lu]Lu-PKB2, likely due to the neutral charged [^177^Lu]-DOTA complex, which most likely increases its lipophilicity and thus the non-specific liver uptake (Hosseinimehr et al. 2012; Rinne et al. 2019; Westerlund et al. 2016). However, [^177^Lu]Lu-PKB3 still showed negligible activity uptake in the liver with < 0.5%IA/g at 2 h pi and fast clearance, minimizing concerns for hepatotoxicity.

Biodistribution studies of [^177^Lu]Lu-AU-RM26-M2 with Entresto pretreatment still showed low tumor uptake probably due to its lower binding affinities (Kanellopoulos et al. 2025). On the contrary, the increased GRPR affinity and in vivo stability of [^177^Lu]Lu-PKB2 and [^177^Lu]Lu-PKB3 resulted in respectively 1.5- and twofold higher activity uptake in tumors compared to [^177^Lu]Lu-AU-RM26-M2. Importantly, these improved tumor targeting properties were achieved without the need of Entresto pretreatment. Additionally, the increased activity uptake in kidneys for [^177^Lu]Lu-AU-RM26-M2 and [^177^Lu]Lu-PKB2 is less corelated with their in vivo stability (Fig. 4) and more with their metal-chelate, indicating that the conformation and/or the extra negative charge are responsible.

The increased in vivo stability with the higher tumor accumulation and retention without the need for NEP-inhibitors, in combination with the high radiochemical purities and yields achieved during labeling represent advantages for future clinical translation.

Notable comparisons can also be made with the preclinical data for clinically validated GRPR-targeting radiopeptides, such as [^177^Lu]Lu-RM2 (RM2: DOTA-Pip-RM26) and its MetTrp^8^-modified analogue [^177^Lu]Lu-AMTG (AMTG: DOTA-Pip-[MetTrp^8^]RM26) (Günther et al. 2022; Dumont et al. 2013). Direct comparison should be made with caution due to differences in experimental protocols, including sampling time points. [^177^Lu]Lu-RM2 showed only 11% intact peptide in murine peripheral blood 30 min pi. The incorporation of MetTrp^8^ in [^177^Lu]Lu-RM2 significantly improved metabolic stability, with 93% intact [^177^Lu]Lu-AMTG at 30 min pi, a similar trend observed in our study. Between 4 and 24 h pi, activity retention in tumors for [^177^Lu]Lu-RM2 decreased to approximately 40%, whereas [^177^Lu]Lu-PKB2 and [^177^Lu]Lu-PKB3 demonstrated high tumor retention of around 80% (Kanellopoulos et al. 2025). For [^177^Lu]Lu-AMTG, only 24 h pi values were reported. Both [^177^Lu]Lu-PKB2 and [^177^Lu]Lu-PKB3 had higher tumor activity uptake 24 h pi than [^177^Lu]Lu-AMTG, although with a slightly higher activity in the kidneys than [^177^Lu]Lu-RM2 and [^177^Lu]Lu-AMTG. Overall, the high in vivo stability, high tumor uptake, and good retention of [^177^Lu]Lu-PKB2 and [^177^Lu]Lu-PKB3 closely align with the performance of [^177^Lu]Lu-AMTG, which is currently undergoing clinical evaluation with no observed side effects (Günther et al. 2022; Kanellopoulos et al. 2025; Dumont et al. 2013). These findings support further evaluation of PKB2 and PKB3, including experimental therapy settings using Lu-177, as well as in combination with other clinically relevant radionuclides (e.g. Ga-68 for PET imaging) or with Ac-225 for the DOTA-carrying peptide for alpha therapy.

## Conclusions

We evaluated two new GRPR-targeting peptides carrying MetTrp^8^ modification in their pharmacophore and compared the use of different chelators: [^177^Lu]Lu-PKB2, bearing a DOTAGA chelator, and [^177^Lu]Lu-PKB3, carrying a DOTA chelator. The incorporation of MetTrp^8^ enhanced the in vivo stability of both peptides, resulting in improved tumor-targeting properties in mice with GRPR-expressing xenografts. The DOTA-carrying peptide showed higher GRPR-affinity and greater PC-3 tumor uptake than its DOTAGA-carrying counterpart, underlining the importance of the local charge and/or complex-conformation at the N-terminus of GRPR-targeting peptides.

## Supplementary Information

Below is the link to the electronic supplementary material.

Supplementary Material 1

## Data Availability

All data generated or analyzed during this study are included in this published article and its supplementary information files.

## Abbreviations

GRPR: Gastrin-releasing peptide receptor
RM26: D-Phe^6^-Gln^7^-Trp^8^-Ala^9^-Val^10^-Gly^11^-His^12^-Sta^13^-Leu^14^-NH_2_
AU-RM26-M2: DOTAGA-PEG_2_-Pip-[Sar^11^]RM26
PEG2: 8-amino-3,6 dioxa-octanoic acid
Pip: 4-amino-1-carboxymethyl-piperidine
MetTrp: α-methyl-L-tryptophan
PKB1: DOTA-PEG_2_-Pip-[Sar^11^]RM26
PKB2: DOTAGA-PEG_2_-Pip-[MetTrp^8^,Sar^11^]RM26
PKB3: DOTA-PEG_2_-Pip-[MetTrp^8^,Sar^11^]RM26
TRT: targeted radionuclide therapy
RM2: DOTA-Pip-RM26
AMTG: DOTA-PEG_2_-Pip-[MetTrp^8^]RM26
NeoB: DOTA-p-aminomethylaniline-diglycolic acid -D-Phe-Gln-Trp-Ala-Val-Gly-His-Leu-NHEt
BBN: bombesin
NEP: neutral endopeptidase (CD10 or neprilisin)
SB3: DOTA-p-aminomethylaniline-diglycolic acid-DPhe-Gln-Trp-Ala-Val-Gly-His-Leu-NHEt
DOTA: 1,4,7,10-tetraazacyclododecane-1,4,7,10-tetraacetic acid
DOTAGA: 1,4,7,10-tetra(carboxymethyl)-1,4,7,10-tetraazacyclo-dodecane-1-glutaric acid
