# Supplementary material for: Improving GRPR-targeting peptides for radiotheranostics application: insights from chelator modifications and α-methyl-L tryptophan substitution
Source: EJNMMI Radiopharm Chem. 2025 Nov 13;10:74. doi: 10.1186/s41181-025-00402-2 (PMC12615863; doi:10.1186/s41181-025-00402-2)
Supplement: Supplementary file 1 — Supplementary Material 1 [file 41181_2025_402_MOESM1_ESM.docx]

**Improving GRPR-targeting peptides for radiotheranostics application: insights from chelator modifications and α-methyl-L tryptophan substitution**

**Karim Obeid^1^, Ekaterina Bezverkhniaia^1^, Vladimir Tolmachev^2^, Anna Orlova^1 3^, Panagiotis Kanellopoulos^1^**

^1^ Department of Medicinal Chemistry, Uppsala University, 751 83 Uppsala, Sweden;

^2^ Department of Immunology, Genetics and Pathology, Uppsala University, 75183 Uppsala, Sweden;

^3^ Science for Life Laboratory, Uppsala University, 752 37 Uppsala, Sweden.

**Supplementary Material**

**
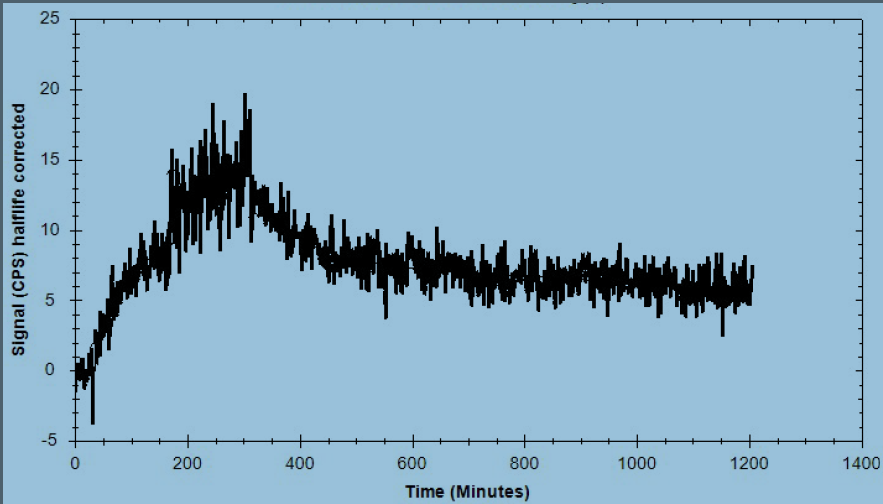
**

**Figure S1: LigandTracer sensorgram for [^177^Lu]Lu-PKB1. For the association measurments two concentrations were used, 1 nM and 5 nM, followed the measurement for the dissociation phase**

**
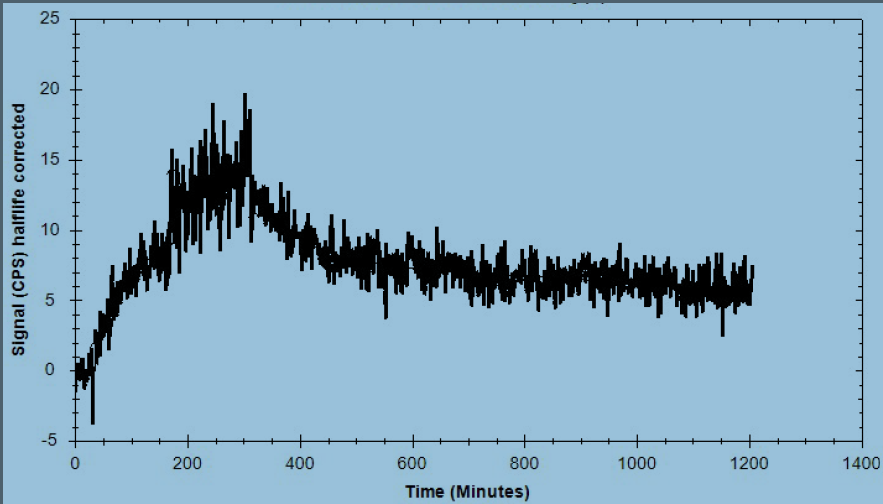
**

**Figure S2: LigandTracer sensorgram for [^177^Lu]Lu-PKB2. For the association measurments two concentrations were used, 1 nM and 5 nM, followed the measurement for the dissociation phase**

**
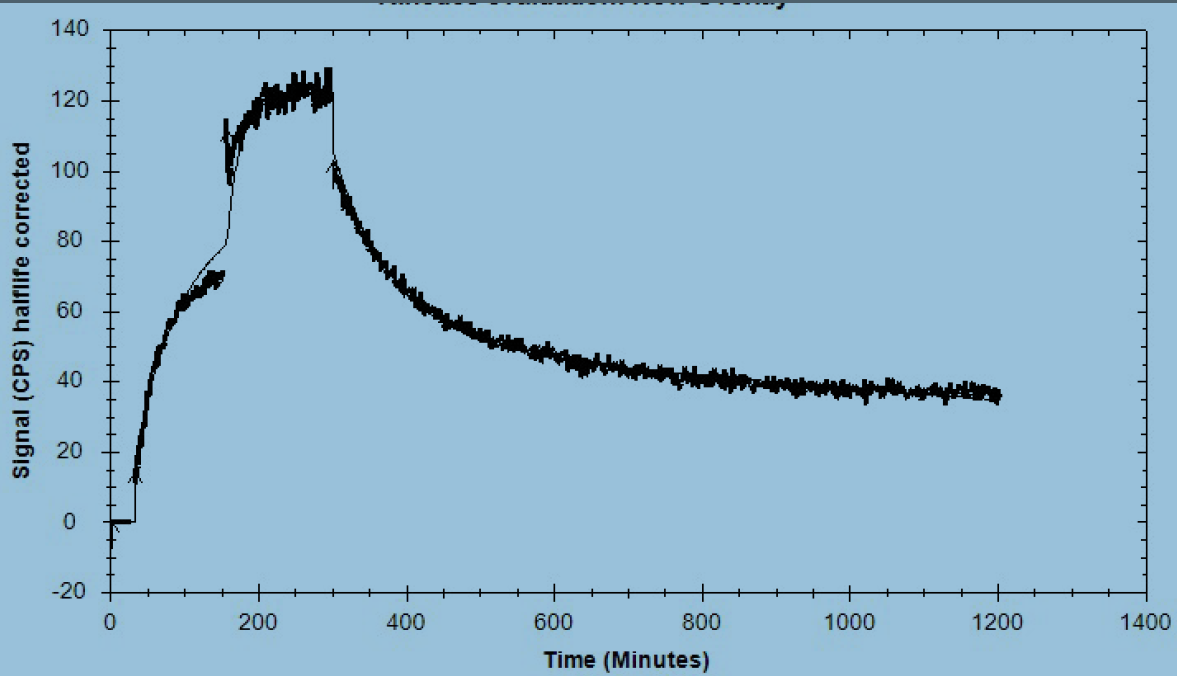
**

**Figure S3: LigandTracer sensorgram for [^177^Lu]Lu-PKB3. For the association measurments two concentrations were used, 1 nM and 5 nM, followed the measurement for the dissociation phase**


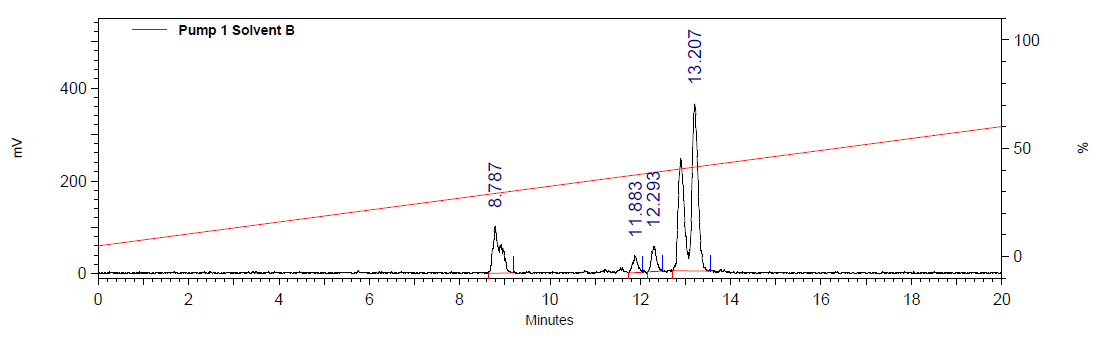


**Figure S4.** Representative radiochromatogram of [^177^Lu]Lu-AU-RM26-M2 in mouse peripheral blood 5 min pi.


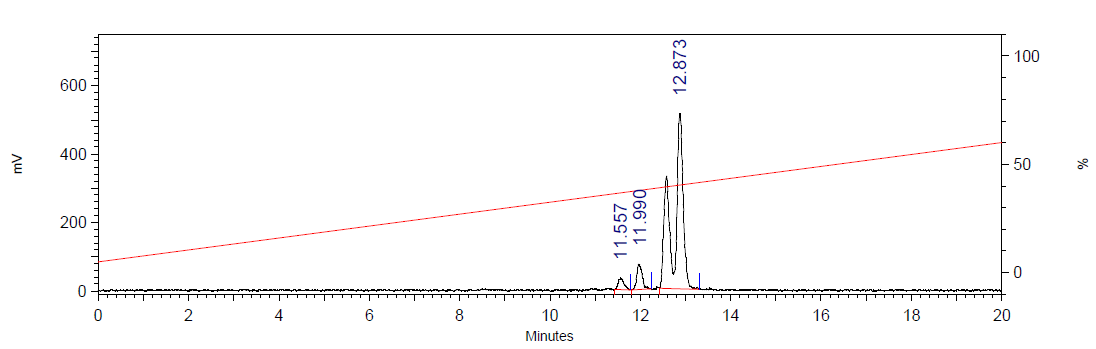


**Figure S5.** Representative radiochromatogram of [^177^Lu]Lu- AU-RM26-M2 in mouse peripheral blood 5 min pi, after pre-treatment with Entresto


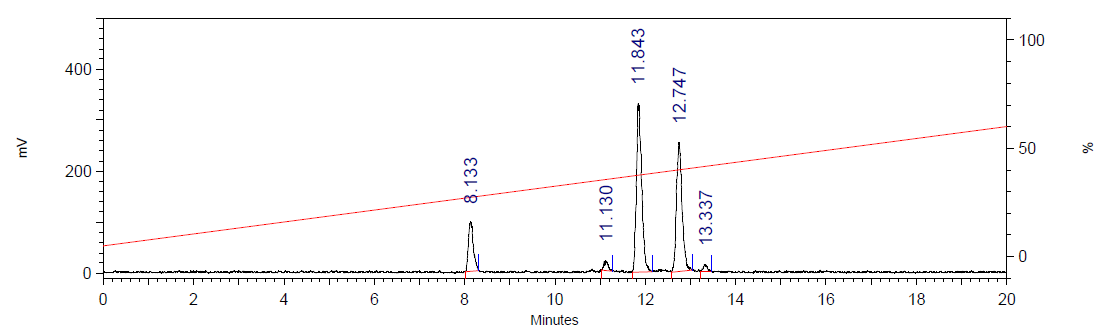


**Figure S6.** Representative radiochromatogram of [^177^Lu]Lu-PKB1 in mouse peripheral blood 5 min pi.


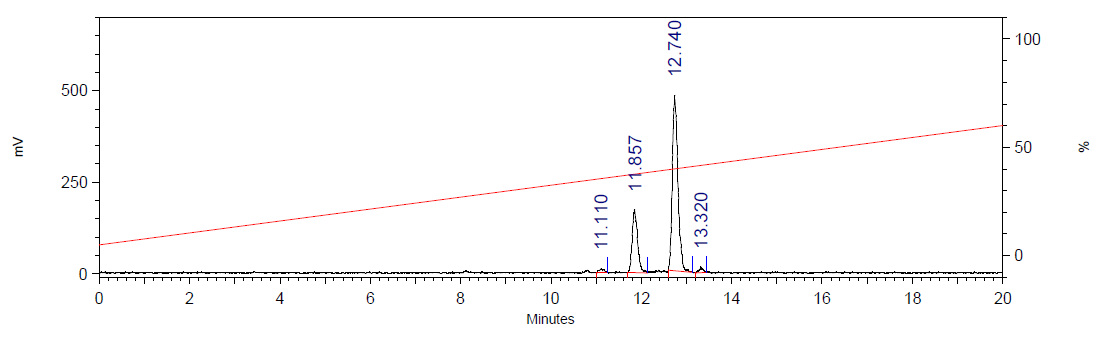


**Figure S7.** Representative radiochromatogram of [^177^Lu]Lu-PKB1 in mouse peripheral blood 5 min pi, after pre-treatment with Entresto.

**
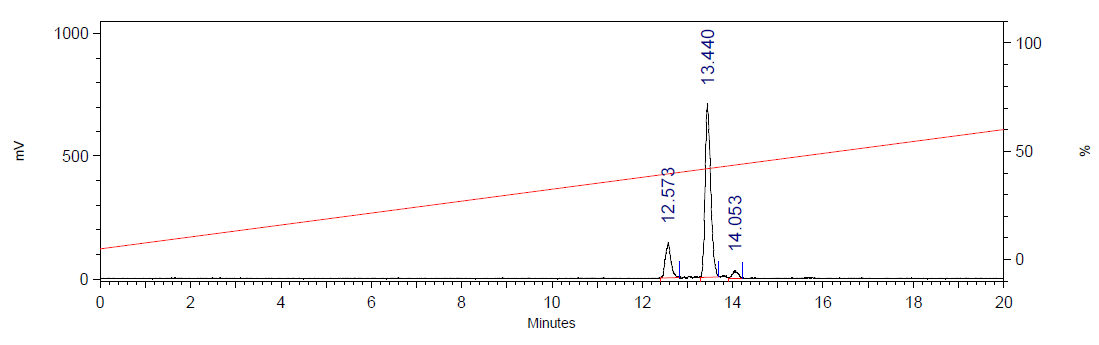
**

**Figure S8.** Representative radiochromatogram of [^177^Lu]Lu-PKB2 in mouse peripheral blood 5 min pi.


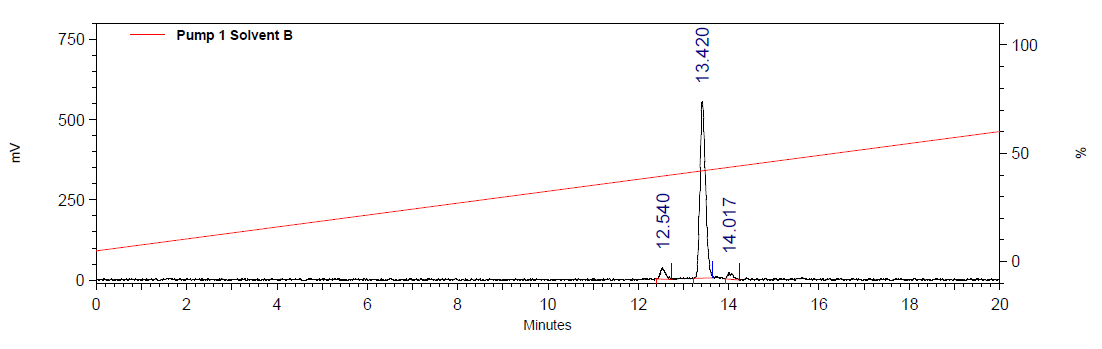


**Figure S9.** Representative radiochromatogram of [^177^Lu]Lu-PKB2 in mouse peripheral blood 5 min pi, after pre-treatment with Entresto.


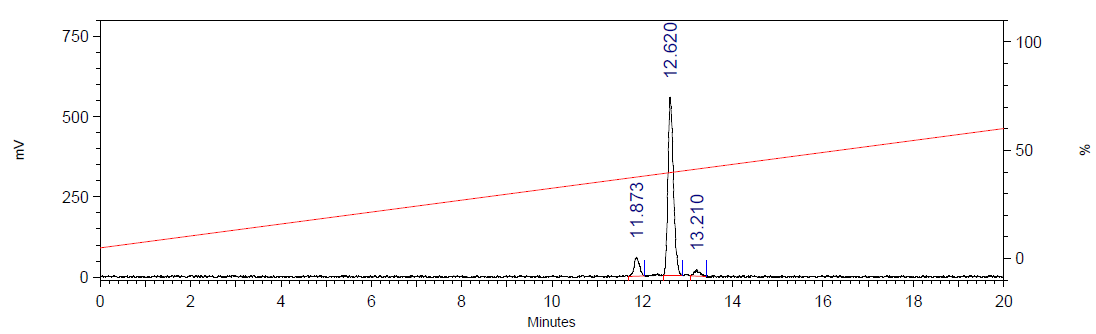


**Figure S10.** Representative radiochromatogram of [^177^Lu]Lu-PKB3 in mouse peripheral blood 5 min pi.


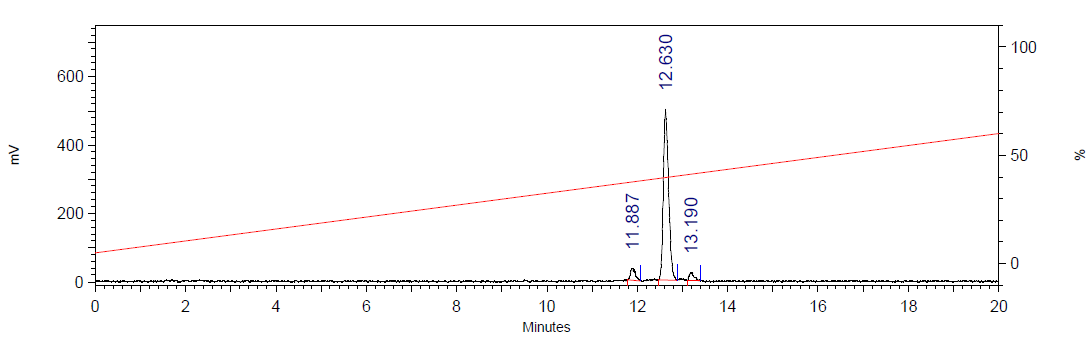


**Figure S11.** Representative radiochromatogram of [^177^Lu]Lu-PKB3 in mouse peripheral blood 5 min pi, after pre-treatment with Entresto.

**Table S1.** Percentage of intact peptide detected by radio-HPLC analysis without (control) or after Entresto pretreatment. Results are given as mean and the two recorded values in parenthesis.

|  | **Control** | **Entresto** |
| --- | --- | --- |
| **Compound** | **Mean [n1 – n2]** | **Mean [n1 – n2]** |
| **[^177^Lu]Lu-AU-RM26-M2*** | 76% [77% - 75%] | 92% [89% - 95%] |
| **[^177^Lu]Lu-PKB1** | 44% [38% - 51%] | 69% [67% - 72%] |
| **[^177^Lu]Lu-PKB2** | 85% [81% - 90%] | 93% [92% - 94%] |
| **[^177^Lu]Lu-PKB3** | 91% [88% - 94%] | 90% [90% - 90%] |
| * Values are taken from previously published data (25). | | |

**Table S2.** Biodistribution profile of [^177^Lu]Lu-PKB2 2 h (blocked and non-blocked), 4 h, and 24 h pi. Results are presented as mean ± SD.

| **[^177^Lu]Lu-PKB2** | | | | |
| --- | --- | --- | --- | --- |
| **Tissue (%IA/g)** | **2 h pi (blocked)**  **[n = 3]** | **2 h pi**  **[n = 4]** | **4 h pi**  **[n = 4]** | **24 h pi**  **[n = 4]** |
| **Blood** | 0.06 ± 0.02 | 0.13 ± 0.05 | 0.07 ± 0.03 | 0.01 ± 0.01 |
| **Lungs** | 0.21 ± 0.03 | 0.25 ± 0.08 | 0.3 ± 0.2 | 0.07 ± 0.01 |
| **Liver** | 0.20 ± 0.02 | 0.24 ± 0.05 | 0.2 ± 0.03 | 0.14 ± 0.02 |
| **Spleen** | 0.12 ± 0.04 | 0.15 ± 0.07 | 0.15 ± 0.06 | 0.11 ± 0.04 |
| **Pancreas** | 1.8 ± 0.3^a^ | 7.3 ± 3.0^a,b,g^ | 2.9 ± 1.0^b,d^ | 0.43 ± 0.09^d,g^ |
| **Stomach** | 0.36 ± 0.07 | 1.8 ± 0.5 | 1.3 ± 0.2 | 0.14 ± 0.07 |
| **Small Int.** | 0.3 ± 0.1 | 0.8 ± 0.5 | 0.5 ± 0.2 | 0.07 ± 0.04 |
| **Kidneys** | 7.6 ± 1.2 | 8.9 ± 2.5g | 9.5 ± 2.1^f^ | 5.5 ± 0.6^f,g^ |
| **Tumor** | 3.1 ± 0.9^a^ | 15.8 ± 3.7^a,g^ | 15.3 ± 1.4^e^ | 12.1 ± 1.3^e,g^ |
| **Muscle** | 0.04 ± 0.01 | 0.07 ± 0.06 | 0.05 ± 0.02 | 0.02 ± 0.01 |
| **Bone** | 0.13 ± 0.03 | 0.13 ± 0.07 | 0.14 ± 0.05 | 0.09 ± 0.04 |
| **GI** | 0.8 ± 0.4 | 1.5 ± 0.4^d^ | 0.7 ± 0.2 | 0.3 ± 0.2 |
| **Body** | 1.7 ± 0.9 | 1.8 ± 1.3 | 2.3 ± 0.8^c^ | 0.5 ± 0.1^c^ |
| Statistical difference between 2 h (blocked) and 2 h pi: p < 0.0001^a^  Statistical difference between 2 h and 4 h pi: p < 0.0001^b^  Statistical difference between 4 h and 24 h pi: p < 0.05^c^p ≤ 0.01^d^, p ≤ 0.001^e^, p ≤ 0.0001^f^  Statistical difference between 2 h and 24 h pi: p ≤ 0.0001^gj^ | | | | |

**Table S3.** Biodistribution profiles of [^177^Lu]Lu-PKB3 2 h (blocked and non-blocked), 4 h, and 24 h pi. Results are presented as mean ± SD1.

| **[^177^Lu]Lu-PKB3** | | | | |
| --- | --- | --- | --- | --- |
| **Tissue (%IA/g)** | **2 h pi (blocked)**  **[n = 3]** | **2 h pi**  **[n = 4]** | **4 h pi**  **[n = 4]** | **24 h pi**  **[n = 4]** |
| **Blood** | 0.13 ± 0.06 | 0.22 ± 0.04 | 0.09 ± 0.02 | 0.02 ± 0.01 |
| **Lungs** | 0.27 ± 0.03 | 0.6 ± 0.3 | 0.24 ± 0.08 | 0.09 ± 0.01 |
| **Liver** | 0.38 ± 0.05 | 0.47 ± 0.05 | 0.42 ± 0.03 | 0.24 ± 0.02 |
| **Spleen** | 0.13 ± 0.02 | 0.4 ± 0.2 | 0.25 ± 0.07 | 0.16 ± 0.06 |
| **Pancreas** | 2.6 ± 0.5^c^ | 11.9 ± 2.1^c,e,k^ | 4.7 ± 0.4^e,h^ | 0.5 ± 0.2^h,k^ |
| **Stomach** | 0.6 ± 0.1^b^ | 2.5 ± 0.2^b,k^ | 1.9 ± 0.3^g^ | 0.16 ± 0.05^g,k^ |
| **Small Int.** | 0.7 ± 0.3^a^ | 2.2 ± 0.6^a,j^ | 1.0 ± 0.3 | 0.11 ± 0.03^j^ |
| **Kidneys** | 5.4 ± 0.7^b^ | 7.4 ± 1.0^b,d^ | 5.9 ± 0.8^d,f^ | 4.3 ± 0.8^f^ |
| **Tumor** | 3.4 ± 0.3^c^ | 23.8 ± 2.5^c,e,k^ | 18.8 ± 0.7^e,h^ | 15.3 ± 2.7^h,k^ |
| **Muscle** | 0.07 ± 0.05 | 0.09 ± 0.02 | 0.1 ± 0.1 | 0.02 ± 0.01 |
| **Bone** | 0.11 ± 0.03 | 0.16 ± 0.03 | 0.13 ± 0.04 | 0.08 ± 0.03 |
| **GI** | 1.0 ± 0.2 | 2.0 ± 0.2^i^ | 0.4 ± 0.3 | 0.16 ± 0.03^i^ |
| **Body** | 1.6 ± 0.7 | 2.7 ± 0.8^i^ | 2.2 ± 0.4 | 1.1 ± 0.7^i^ |
| Statistical difference between 2 h (blocked) and 2 h pi: p < 0.05^a^, p ≤ 0.01^b^, p ≤ 0.0001^c^  Statistical difference between 2 h and 4 h pi: p < 0.05^d^, p ≤ 0.0001^e^  Statistical difference between 4 h and 24 h pi: p < 0.05^f^, p ≤ 0.01^g^, p ≤ 0.0001^h^  Statistical difference between 2 h and 24 h pi: p < 0.01^i^, p ≤ 0.001^j^, p ≤ 0.0001^k^ | | | | |
